# Observations and Reminiscences on the Use of Chloroform and Ether

**Published:** 1859-07

**Authors:** P. Pineo


### ARTICLE XII.

### *Observations and Reminiscences on the Use of Chloroform and Ether.* By P. Pineo, M. D.

It was my good fortune to witness the first capital operation performed under the influence of the *Letheon,* at the Massachusetts General Hospital, in the fall of 1846. Since that time, more than 12 years, I have been in the almost daily habit of administering ether and chloroform, in midwifery, as well as in surgical and medical cases, and my experience has been entirely free from fatal or even very uncomfortable results.

That there is danger in the injudicious use of both ether and chloroform, is not to be questioned. Of the comparative danger of the two, there is also no question—chloroform being more dangerous, not *per se,* but because it requires more care and enlightened experience in watching the effect on the system. It is more rapid in its effects, and requires a larger admixture of the oxygenating influence of the atmosphere, than ether.

That a perfectly healthy animal or human being can be brought to a state of apparent death, by anæsthesia, with comparative safety, I have no question. I have subjected different animals—cats, dogs, mice, &c.—to the influence of chloroform and ether until there was no sign of life, and witnessed their gradual recovery when exposed to atmospheric air; often without any attempt at resuscitation, though sometimes artificial respiration was required. I have given to horses sufficient chloroform to control all motion, and in one case operated for cataract on both eyes, without the slightest twitching of a muscle, both eyes being fixed and immovable. In another case, I kept a horse near two hours under the influence of chloroform, in performing a prolonged operation. Perfect recovery was soon manifest when pure air was allowed.

When chloroform was first introduced, I was called to attend a woman in labor with her first child. The patient was 22 years of age, finely proportioned, fat, and enjoying rude or rustic health. The child proved to be unusually large, weighing 14 pounds and some ounces. The labor was long continued, and difficult. At a proper stage of the labor, I administered chloroform freely, short only of stopping the pains. Other patients requiring my attention, I left the woman for a short time, and was unavoidably kept away two or three hours. I left several ounces of chloroform, giving permission to the nurse to use it carefully if the patient suffered greatly. On my return, I found that my chloroform had disappeared, and from accounts, the patient had been kept insensible by the use of it for hours. The patient was delivered without instruments, and had a quick and perfect recovery.

A young man, about 21 years of age, very muscular, possessing the same rude and robust health as the preceding patient, has had strangulated inguinal hernia, three or four times at different periods within about two years. Extreme muscular prostration was necessary in order to reduce the hernia. Chloroform was given to complete relaxation and apparent death, the tongue falling back over the glottis, and all respiration ceasing, and even the heart's action being undiscernible. By discontinuing the chloroform, seizing the tongue and pulling it forward, exposing the patient to fresh air, and exciting artificial respiration by the postural method, the patient would soon resume his breathing, and the next day be quite comfortable.

But there are cases with a low and anæmic condition of the system, with or without cardiac disease, where the greatest caution is necessary. I have recently had two or three such cases. On inhaling a small quantity of a mixture of chloroform and ether, great irregularity of the heart became manifest, a disposition to syncope, a nameless distress, &c. In one case, these symptoms were present when the patient had not the slightest degree of fear about breathing the anæsthetic.

Mrs. W. had been subject for years to occasional attacks of rheumatism and irregular action of the heart. My diagnosis was organic disease of the heart. Having decayed and troublesome teeth, and wishing them extracted, she requested me to go with her to the dentist and administer an anæsthetic. She would not be dissuaded, but insisted quite peremptorily on inhalation, and I acquiesced in her urgent wish, and administered a mixture of ether and chloroform. I carefully watched the effect on the heart, desisting as soon as untoward symptoms were manifest, but etherizing sufficiently for the patient to have several teeth extracted, and almost without pain. The next day the patient felt no unfavorable effect from the inhalation. A few months after, she died in an instant from disease of the heart.

The foregoing cases tend to show:

1st—That perfectly healthy and robust human beings and animals can breathe chloroform and ether, to almost any reasonable extent, with impunity.

2d—That anemic patients, with a depressed condition of the system, flabby heart, &c., require great care and watchfulness during the administration of anæsthetics; and although we cannot always fully diagnosticate these cases, yet, with caution, and a knowledge which can appreciate danger in its incipiency, with the prompt use of stimulants and restoratives when necessary, we can generally insure against accidents in such cases.

3d—That the fact of structural disease of the heart, being present need not wholly exclude the use of anæsthesia, but it should in such cases be resorted to with great caution.

For an inhaler, I use a folded napkin, which permits the ready admixture of the atmosphere to whatever extent may be desired.

For slight operations, I think the rule should be, the minimum dose that will produce the effect desired.

The quality of the chloroform and ether is a most important consideration; too much care to have them pure, cannot be taken. The impurity of the material has often much to do with the uncomfortable effects produced.

*Queechy, Vt., March* 21, 1859.

[*Boston Med. and Surg. Jour.*

---

